# Alltagsbeeinträchtigungen bei neurokognitiven Störungen: Entwicklung eines differenzierten Erfassungsinstrumentes für Forschung und Praxis

**DOI:** 10.1007/s00115-021-01257-z

**Published:** 2022-01-10

**Authors:** Katja Funke, Marie Bernard, Melanie Luppa, Steffi G. Riedel-Heller, Tobias Luck

**Affiliations:** 1 Institut für Sozialmedizin, Rehabilitationswissenschaften und Versorgungsforschung (ISRV) & Fachbereich Wirtschafts- und Sozialwissenschaften, Hochschule Nordhausen, Nordhausen, Deutschland; 2 Institut für Gesundheits- und Pflegewissenschaft, Medizinische Fakultät, Martin-Luther-Universität Halle-Wittenberg, Halle (Saale), Deutschland; 3 Institut für Medizinische Soziologie, Medizinische Fakultät, Martin-Luther-Universität Halle-Wittenberg, Halle (Saale), Deutschland; 4 SRH Hochschule für Gesundheit, Gera, Deutschland; 5 Institut für Sozialmedizin, Arbeitsmedizin und Public Health (ISAP), Medizinische Fakultät, Universität Leipzig, Leipzig, Deutschland; 6 Fakultät Angewandte Sozialwissenschaften, Fachhochschule Erfurt, Altonaer Str. 25, 99085 Erfurt, Deutschland

## Hintergrund

Bei der Diagnostik von Demenzen und deren potenziellen Frühformen kommt der sorgfältigen Erfassung, inwieweit betroffene Menschen ihren Alltag noch bzw. nicht mehr bewältigen können, eine enorme Bedeutung zu. So müssen beispielsweise für die Diagnose einer Demenz nach den Kriterien des internationalen Klassifikationssystems International Classification of Diseases—10th Revision (ICD-10) mit einem objektiven kognitiven Abbau einhergehende Beeinträchtigungen von Aktivitäten des täglichen Lebens nachgewiesen werden [4]. Die aktuelle fünfte Version des Klassifikationssystems Diagnostic and Statistical Manual of Mental Disorders (DSM) unterscheidet explizit zwischen einer *schweren neurokognitiven Störung* – eine Diagnose, die das noch im DSM-IV aufgeführte Demenzsyndrom in etwa ersetzt – und einer *leichten neurokognitiven Störung*, welche in vielen Fällen eine mögliche Vorstufe/frühe Ausprägung einer Demenz darstellen kann [1, 2]. Auch für die Diagnose dieser beiden Formen neurokognitiver Störungen und insbesondere deren Abgrenzung voneinander stellen Beeinträchtigungen in den Alltagsaktivitäten ein entscheidendes Kriterium dar, müssen für die schwere neurokognitive Störung doch eine durch die kognitiven Einschränkungen beeinträchtigte Unabhängigkeit in der Verrichtung alltäglicher Aktivitäten nachgewiesen werden, während bei der leichten neurokognitiven Störung die selbständige Verrichtung trotz kleinerer Einschränkungen noch möglich sein sollte [2]. Auch mit der in der neuen 11. Version der ICD-Klassifikation ebenfalls vorgenommenen Aufnahme der Diagnose *leichte neurokognitive Störung* [5] wird die Bedeutung der sorgfältigen Erfassung von Beeinträchtigungen in Alltagsaktivitäten zunehmen. Trotz dieser enormen Relevanz von Alltagsbeeinträchtigungen wird Diagnostiker/innen bei deren Erfassung erstaunlich viel Spielraum überlassen.

## Zielsetzung

Ziel des hier vorgestellten und von der Deutschen Alzheimer Gesellschaft e. V. – Selbsthilfe Demenz geförderten Forschungsprojektes „Entwicklung eines Instrumentes für die differenzierte Erfassung von Alltagsbeeinträchtigungen aufgrund kognitiver Abbauprozesse – Ein Ansatz zur Verbesserung der Früherkennung und Diagnostik von Demenzen und deren Vorstufen leichter neurokognitiver Störungen in Forschung und Praxis“ ist die Entwicklung, Evaluation und Bereitstellung eines standardisierten deutschsprachigen Instrumentes für die differenzierte, ökonomische Erfassung von Alltagsbeeinträchtigungen aufgrund neurodegenerativer Abbauprozesse. Das Erfassungsinstrument soll eine empirisch begründete Differenzierung von Alltagsbeeinträchtigungen ermöglichen, welche

1. mit leichten neurokognitiven Störungen und schweren neurokognitiven Störungen/Demenzen assoziiert sind (Differenzierung nach dem Schweregrad) und
2. mit Defiziten in spezifischen kognitiven Bereichen assoziiert sind (entsprechend der sechs im DSM‑5 differenzierten neurokognitiven Domänen [2]; Differenzierung nach dem Bereich der kognitiven Defizite).

Die Bereitstellung des Erfassungsinstrumentes soll die Früherkennung und (Differenzial‑)Diagnostik von Demenzen sowie deren (potenziellen) Vorstufen in Forschung und klinischer Praxis unterstützen und damit zur Verbesserung der Versorgungssituation der Betroffenen selbst sowie auch zu einer Entlastung deren Angehöriger beitragen.

## Methodik

Das Forschungsprojekt wird in Kooperation der Hochschule Nordhausen (Prof. Dr. rer. med. habil. Tobias Luck, Principal Investigator) mit der Universität Leipzig (Prof. Dr. med. Steffi G. Riedel-Heller, MPH, Kooperationspartnerin) durchgeführt und umfasst zwei Projektphasen.

In der an der Hochschule Nordhausen durchgeführten *Projektphase 1* (01.03.2020–30.04.2021) erfolgte die Erstellung einer Pilotversion des Erfassungsinstrumentes. Hierfür wurde zunächst in drei sich einander ergänzenden Arbeitsschritten ein umfassender potenzieller Itempool für das Instrument generiert:

1. Literaturrecherche in (inter-)nationalen Fachdatenbanken,
2. Analyse bereits existierender Untersuchungsinstrumente,
3. Durchführung einer Expert/innenbefragung (standardisierte postalische Befragung von *n* = 20 klinisch tätigen Diagnostiker/innen).

**Figure 1:**
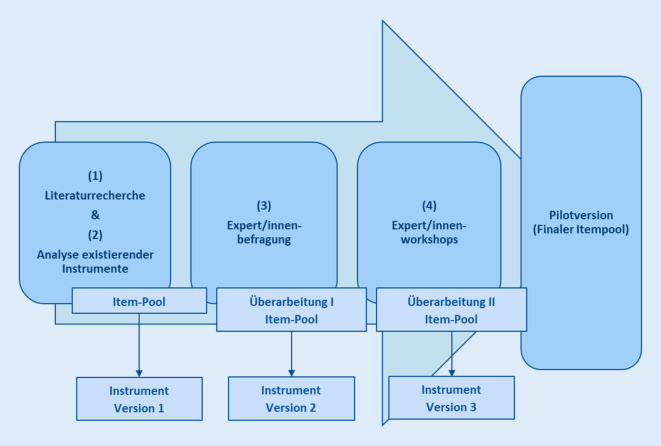

Aufbauend auf dem erarbeiteten Itempool erfolgte in einem 4. Arbeitsschritt im Rahmen von acht Expert/innenworkshops die finale Erstellung der Pilotversion des Instrumentes (Abb. 1). An den Workshops nahmen insgesamt *n* = 24 Personen aus vier Gruppen (*n* = 6 Senior/innen, *n* = 6 Angehörige älterer Menschen mit Demenz, *n* = 7 Pflege- und Gesundheitsfachkräfte, *n* = 5 Diagnostiker/innen) teil. In den Workshops wurde eine Vorversion des Instrumentes auf Anwendbarkeit, Vollständigkeit und Verständlichkeit geprüft. Hierzu wurden von zwei Projektmitarbeiter/innen anhand eines leitfadenbasierten, halbstandardisierten Interviews Fragen zu Instruktion, Items sowie der Antwortskala des Erfassungsinstrumentes gestellt und ausgewertet. Die Durchführung der Workshops erfolgte aufgrund der Corona-Pandemie ausschließlich online per Videokonferenzsystem BigBlueButton (Open-Source-Webkonferenzsystem; https://bigbluebutton.org/).

In der aktuell an der Universität Leipzig laufenden *Projektphase 2* (01.03.2021–31.08.2022) erfolgt die psychometrische Evaluierung und Finalisierung des Erfassungsinstrumentes im Rahmen einer Pilotstudie mit insgesamt *n* = 90 Proband/innen (60+ Jahre) unterteilt in drei Gruppen: *n* = 30 Proband/innen ohne kognitive Defizite, *n* = 30 Proband/innen mit leichten neurokognitiven Störungen sowie *n* = 30 Proband/innen mit klinisch manifester Demenz. Detaillierte Ziele der Pilotstudie sind neben der Überprüfung der Verständlichkeit und Klarheit des Erfassungsinstrumentes die Überprüfung der psychometrischen Gütekriterien [3] und eine weitere Reduktion der Itemanzahl.

Alle beschriebenen Befragungen und Untersuchungen von Studienteilnehmer/innen erfolgten bzw. erfolgen mit Zustimmung der zuständigen Ethikkommissionen (Projektphase 1: Ethikkommission des Universitätsklinikums Jena, AZ 2020-1709-Bef & 2020-1709_1-Bef; Projektphase 2: Ethikkommission an der Medizinischen Fakultät der Universität Leipzig, AZ 217/21-ek) im Einklang mit nationalem Recht sowie gemäß der Deklaration von Helsinki von 1975 (in der aktuellen, überarbeiteten Fassung; [6]). Von allen beteiligten Studienteilnehmer/innen liegt eine Einverständniserklärung vor bzw. wird eine Einverständniserklärung vor Teilnahme eingeholt.

## Aktuelle Ergebnisse

**Figure 2:**
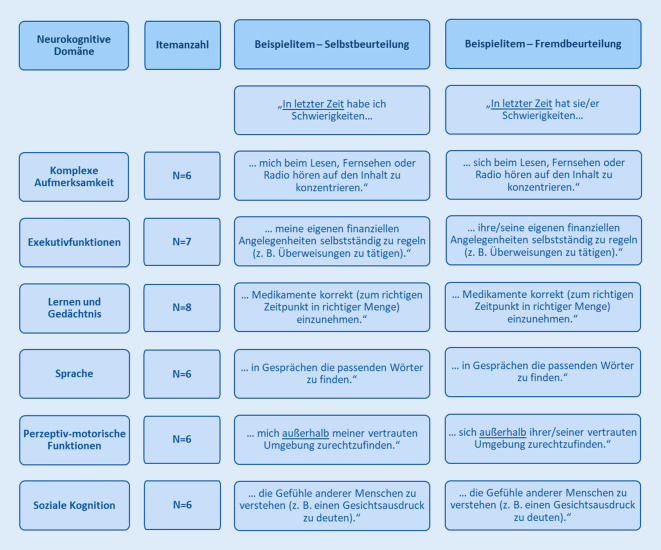

Mit dem Abschluss der Projektphase 1 konnte eine Pilotversion des *Instrumentes für die Erfassung von Alltagsbeeinträchtigungen bei neurokognitiven Störungen (A-NKS)* erstellt werden, die aktuell in Projektphase 2 evaluiert wird. Die Pilotversion wurde hierbei sowohl als Selbst- (A-NKS-SB) als auch als Fremdbeurteilungsversion (A-NKS-FB) erstellt. Beide Versionen umfassen jeweils 39 Items, die, den Zielen des Forschungsprojektes entsprechend, so zusammengestellt wurden, dass sie Beeinträchtigungen in Alltagsaktivitäten erfassen sollen, die Defiziten in den vom DSM‑5 differenzierten sechs neurokognitiven Domänen [2] spezifisch zugeordnet werden können. Einen Überblick über die neurokognitiven Domänen des DSM‑5, die Anzahl der jeweils in der erstellten Pilotversion des Erfassungsinstrumentes zugeordneten Items sowie Beispielitems aus Selbst- und Fremdbeurteilungsversion gibt Abb. 2. Zur Beurteilung des Vorliegens der Alltagsbeeinträchtigungen wurde in den beiden Versionen allen Items eine Antwortskala zugeordnet (keine Schwierigkeiten – leichte Schwierigkeiten – mittlere Schwierigkeiten – große Schwierigkeiten – Es gelingt mir/ihr/ihm gar nicht – Trifft auf mich/sie/ihn nicht zu), welche zusätzlich eine Differenzierung nach dem Schweregrad einer potenziell vorliegenden neurokognitiven Störung/Demenz erlauben soll.

## Ausblick

Nach erfolgreicher Evaluierung und Finalisierung der Pilotversion des Erfassungsinstrumentes in Projektphase 2 wird die finale Version einem breiten Anwender/innenkreis in Forschung und Praxis (Hausärzt/innen, Fachärzt/innen, Neuropsycholog/innen etc.). kostenfrei zur Verfügung gestellt.

## Fazit für die Praxis

- Es wird ein Erhebungsinstrument entwickelt und kostenfrei zur Verfügung gestellt, welches eine Erfassung von Alltagsbeeinträchtigungen bei neurokognitiven Störungen differenziert nach dem Schweregrad (leichte vs. schwere neurokognitive Störung/Demenz) und nach dem Bereich der kognitiven Defizite (entsprechend den sechs neurokognitiven Domänen des DSM-5) ermöglicht.
- Das Instrument soll so die Früherkennung und (Differenzial‑)Diagnostik von Demenzen sowie deren (potenziellen) Vorstufen in Forschung und klinischer Praxis unterstützen.
